# Active auroral arc powered by accelerated electrons from very high altitudes

**DOI:** 10.1038/s41598-020-79665-5

**Published:** 2021-01-18

**Authors:** Shun Imajo, Yoshizumi Miyoshi, Yoichi Kazama, Kazushi Asamura, Iku Shinohara, Kazuo Shiokawa, Yoshiya Kasahara, Yasumasa Kasaba, Ayako Matsuoka, Shiang-Yu Wang, Sunny W. Y. Tam, Tzu‑Fang Chang, Bo‑Jhou Wang, Vassilis Angelopoulos, Chae-Woo Jun, Masafumi Shoji, Satoko Nakamura, Masahiro Kitahara, Mariko Teramoto, Satoshi Kurita, Tomoaki Hori

**Affiliations:** 1 Institute for Space-Earth Environmental Research, Nagoya University, Furo-cho, Chikusa-ku, Nagoya, Aichi 464-8601 Japan; 2 Academia Sinica Institute of Astronomy and Astrophysics, 11F Astronomy-Mathematics Building, AS/NTU, No. 1, Sec. 4, Roosevelt Road, Taipei, 10617 Taiwan; 3 Institute of Space and Astronautical Science, Japan Aerospace Exploration Agency, 3-1-1 Yoshinodai, Chuo-ku, Sagamihara, Kanagawa 252-5210 Japan; 4 Graduate School of Natural Science and Technology, Kanazawa University, Kakuma-machi, Kanazawa, Ishikawa 920-1192 Japan; 5 Planetary Plasma and Atmospheric Research Center, Tohoku University, 6-3 Aoba, Aramaki, Aoba-ku, Sendai, Miyagi 980-8578 Japan; 6 Data Analysis Center for Geomagnetism and Space Magnetism, Graduate School of Science, Kyoto University, Oiwake-cho, Kitashirakawa, Sakyo-ku, Kyoto, Kyoto 606-8502 Japan; 7 Institute of Space and Plasma Sciences, National Cheng Kung University, No.1, University Road, Tainan, 70101 Taiwan; 8 Department of Earth, Planetary, and Space Sciences, University of California, 595 Charles Young Drive East, Los Angeles, CA 90095-1567 USA; 9 Faculty of Engineering, Kyushu Institute of Technology, 1-1 Sensui-cho, Tobata-ku, Kitakyushu, Fukuoka 804-8550 Japan; 10 Research Institute for Sustainable Humanosphere, Kyoto University, Gokasho, Uji, Kyoto 611-0011 Japan

**Keywords:** Aurora, Magnetospheric physics

## Abstract

Bright, discrete, thin auroral arcs are a typical form of auroras in nightside polar regions. Their light is produced by magnetospheric electrons, accelerated downward to obtain energies of several kilo electron volts by a quasi-static electric field. These electrons collide with and excite thermosphere atoms to higher energy states at altitude of ~ 100 km; relaxation from these states produces the auroral light. The electric potential accelerating the aurora-producing electrons has been reported to lie immediately above the ionosphere, at a few altitudes of thousand kilometres^1^. However, the highest altitude at which the precipitating electron is accelerated by the parallel potential drop is still unclear. Here, we show that active auroral arcs are powered by electrons accelerated at altitudes reaching greater than 30,000 km. We employ high-angular resolution electron observations achieved by the Arase satellite in the magnetosphere and optical observations of the aurora from a ground-based all-sky imager. Our observations of electron properties and dynamics resemble those of electron potential acceleration reported from low-altitude satellites except that the acceleration region is much higher than previously assumed. This shows that the dominant auroral acceleration region can extend far above a few thousand kilometres, well within the magnetospheric plasma proper, suggesting formation of the acceleration region by some unknown magnetospheric mechanisms.

## Introduction

Discrete auroral arcs are a manifestation of energy flow from the magnetosphere to the ionosphere^1^. Auroral light is excited by ~ 1–10 keV electrons precipitating into the ionosphere while carrying an upward field-aligned current—a portion of the magnetosphere–ionosphere current system. Since most magnetospheric electrons cannot reach the ionosphere because of the magnetic mirror force, an electric field that accelerates electrons downward (parallel to the magnetic field) is usually required to satisfy the need for current closure and ensure current continuity^2,3^. This parallel electric field has been directly measured in-situ in the so-called acceleration region^4,5^ and also confirmed by other parallel acceleration signatures^6–9^ but the generation mechanism of the parallel electric field remains unknown. This mechanism is important for auroral physics and magnetosphere–ionosphere coupling in Earth’s polar ionosphere and other the planetary magnetospheres such as for Jupiter. However, even the altitude extent of this acceleration region is not presently conclusive; thus important observational constraints on its likely generation mechanism are lacking.

A typical representation of the acceleration region observed by previous low altitude satellites is as follows^6^. Downgoing monoenergetic electrons, whose flux often forms the inverted-V shape in an energy-time spectrogram, indicate that these electrons are accelerated above a satellite prior to precipitating to auroral altitudes. These precipitating electrons could be the main carriers of the upward field-aligned current, which is observed as an azimuthal magnetic field deflection. In the region of those accelerated current carriers, the plasma number density, also known as plasma cavity, is expected to be small. The upgoing proton and converging electric field indicate a U-shape potential drop below a satellite. The lack of upgoing electrons within the loss cone is consistent with accelerated electrons that are lost to the atmosphere while exciting auroral emissions. Since the parallel acceleration makes a pitch angle more field-aligned, in contrast to the mirror force, the electron loss cone width depends on the potential drop below a satellite. Such an acceleration region is assumed to lie in the transition region between ionospheric and magnetospheric plasmas, typically at 1000–20,000 km altitudes^1^. However, there have been several reports of signatures reminiscent of potential-driven acceleration above 20,000 km, outside the ionosphere-magnetosphere transition region^7–10^. It is unclear whether electrons accelerated from such high altitudes can precipitate to the ionosphere and excite auroral emissions, owing to the small loss cone in the region. The high-angular-resolution electron detector equipped on the Arase satellite^11^ can resolve such a small loss cone, and to the best of our knowledge, there is no observation of the loss cone of electrons accelerated by the potential drop at such high altitudes. Additionally, THEMIS (Time History of Events and Macroscale Interactions during Substorms) ground-based all-sky imagers^12,13^ have sufficiently high temporal and spatial resolutions, which were not to coordinate with past, the previous high-altitude satellite missions. This study utilizes this unique opportunity to investigate the auroral acceleration properties at high altitudes using comprehensive particle and field observations (including high-angular resolution electron observations)^11,14–18^ with the Arase satellite^19^ and the network of THEMIS ground-based imagers.

## Results and discussion

**Figure 1:**
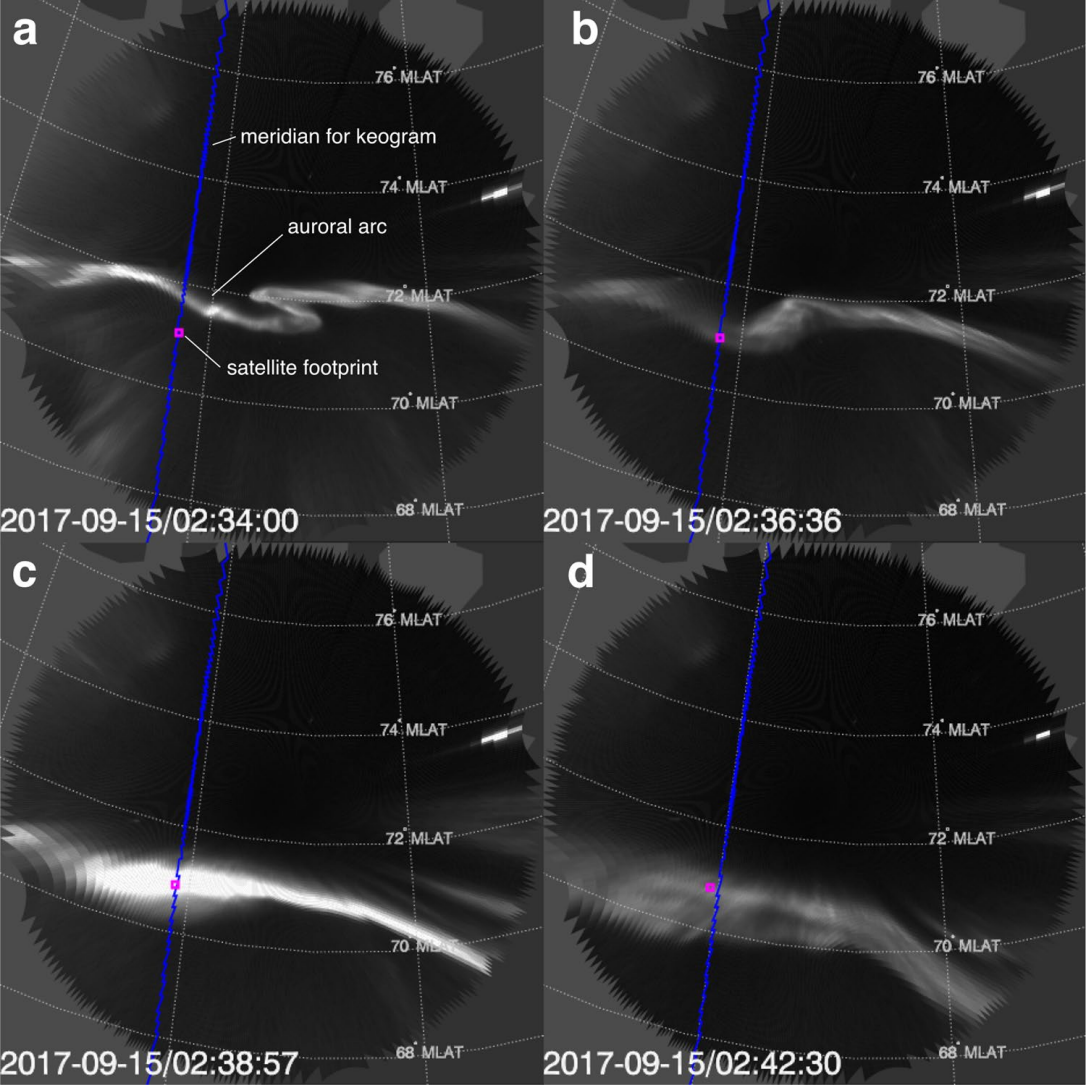
Images of the auroral arc around the footprint of Arase. The images were captured at 02:34:00–02:42:30 UT on 15 September, 2017 by the all-sky camera at Rankin Inlet (RANK). The magenta square shows the ionospheric footprint of Arase determined by the Tsyganenko and Andreeva (TA) 15B model. The latitudinal grid shows the magnetic latitude in altitude-adjusted corrected geomagnetic coordinates. The blue line shows the meridian along which the south-to-north cross-sectional time series of auroral intensity were collected for Fig. 2. Supplementary Video S1 shows the full motion of the auroral arc at a 3-s cadence.

On 15 September, 2017, 02:35–02:42 UT, Arase was at an altitude of ~ 30,000 km, ~ 38° dipole latitude, and ~ 20 h magnetic local time. Arase was magnetically mapped to a thin active auroral arc; the evolution of the arc was captured by the THEMIS all-sky imager at Rankin Inlet (RANK) (Fig. 1 and Supplementary Video S1). As the arc moved equatorward, Arase’s footprint moved poleward and into the arc (Fig. 1a,b), where it remained for the next 7 min; subsequently, it exited slowly thereafter from the poleward side of the arc (Fig. 1c,d). During the 7-min period, the arc brightened (Fig. 1c), then dimmed and broadened in latitude (Fig. 1d). Ionospheric footprints estimated by several state-of-art magnetic field models^20–23^ show that the satellite was likely in the flux tube of the arc (Supplementary Fig. S1). This arc variation occurred during the recovery phase of an auroral substorm^24^, when Arase was located in the plasma sheet boundary layer (PSBL), the transition layer between the plasma sheet and the magnetotail lobe (Supplementary Fig. S2).

**Figure 2:**
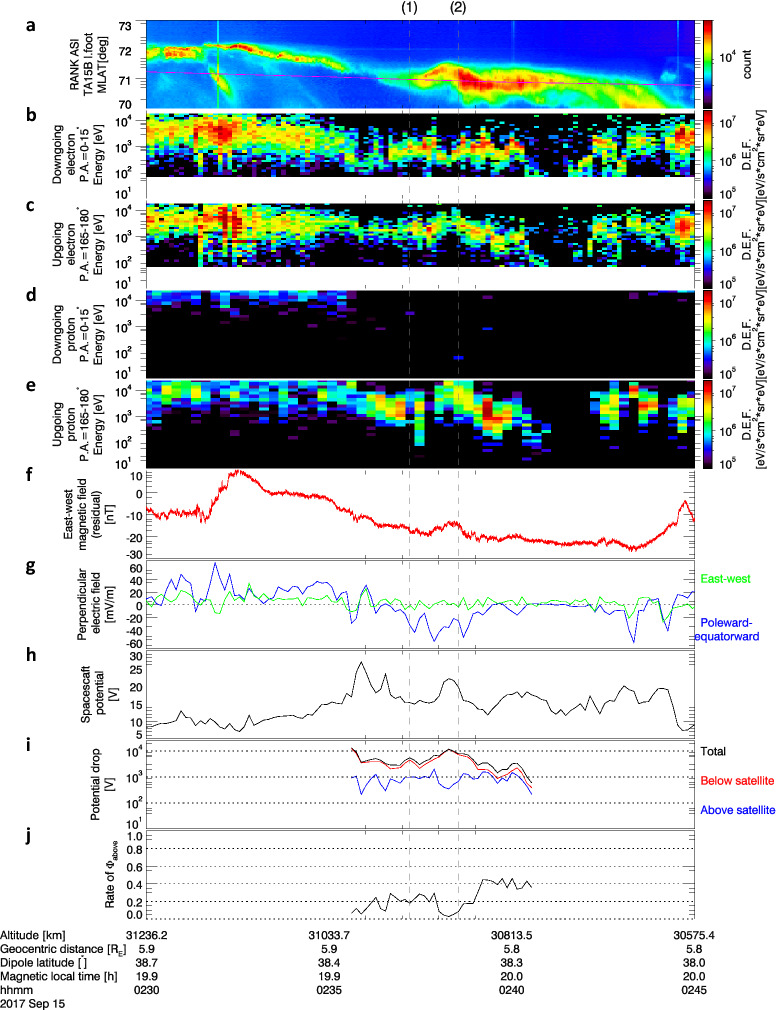
Time series data of observations by Arase. (**a**) Auroral intensity along the south-to-north cross-section at the blue line shown in Fig. 1. Magenta line indicates the magnetic latitude of Arase’s footprint. Differential energy fluxes of: (**b**) downgoing electrons at the pitch angle of 0°–15°; (**c**) upgoing electrons at the pitch angle of 165°–180°; (**d)** downgoing protons at the pitch angle of 0°–15°; and (**e**) upgoing protons at the pitch angle of 165°–180°. The white region indicates no data in the pitch angle range. (**f**) Residual east–west magnetic field (azimuthal direction with respect to the Earth’s dipole axis) subtracted by quiet time magnetic field of Tsyganenko 89 model^36^ at the *Kp* index of 0. (**g**) Electric field perpendicular to the magnetic field. Blue and green represent poleward and eastward electric fields, respectively. (**h**) Spacecraft potential as a proxy of electron density. (**i**) Estimated potential drops above and below the satellite. (**j**) Ratio of potential drop above the satellite to the total potential drop. The vertical dashed lines show times when phase space density distributions are shown for Fig. 3.

Potential-driven acceleration powered the auroral arc; this is depicted by the time series of auroral intensity at the Arase footprint (Fig. 2a) as evidenced by the following: first, monoenergetic downgoing electrons at Arase (accelerated above the satellite) show peak flux energy ~ 1 keV for the period 02:36–02:41 UT (Fig. 2b); second, the upgoing electron flux for the same period is lower than the downgoing electron flux (Fig. 2c), bespeaking of a net upward field-aligned current; third, upgoing monoenergetic protons with energy of 1–10 keV (accelerated below the satellite) are present, while there are very few downgoing protons (Fig. 2d,e), implying that the potential acceleration (upward electric field) region also extends below Arase’s altitude.

The east–west magnetic field component variation (Fig. 2f, positive eastward) exhibited a negative deflection (westward) during Arase’s poleward motion across the arc (02:35–02:42UT), indicating traversal of an upward field-aligned current sheet. The electric field perpendicular to the magnetic field is strongly polarized in the latitudinal direction, indicating that the flux tube on the east–west aligned arc is the source of the electric field (Fig. 2g, positive poleward and eastward). The electric field was directed poleward when the satellite was located on the equatorward side of the arc, whereas the field was directed equatorward as the arc approached. Thus, the radial perpendicular electric field converged near the arc, demonstrating the U-shape potential drop in the flux tube of the arc. The spacecraft potential, inversely proportional to the logarithm of the electron plasma density, increased after 02:34 UT and peaked at 02:36 UT (Fig. 2h). The implied decrease in the electron density (a so-called density cavity) is a typical signature of the auroral acceleration region.

The potential drop is estimated both above and below the satellite using the particle data^25,26^ for the period 02:35:36–02:40:40 UT, which the unidirectional upgoing ions appeared. If the parallel kinetic energy of charged particles originates from acceleration by the parallel electric field, the energy divided by the electric charge can be interpreted as a potential drop. Here we consider the mean energy per particle, the ratio of the energy flux to the number flux, as energy given by a potential drop (Fig. 2i). The potential drop above the satellite estimated from the downgoing electrons is ~ 1 kV while that below the satellite estimated from the upgoing protons is ~ 1–10 kV, and these vary independently. Despite the high altitude of the satellite, the potential drop above the satellite it is often a significant fraction of the total drop, as large as 20–45% (Fig. 2j).

**Figure 3:**
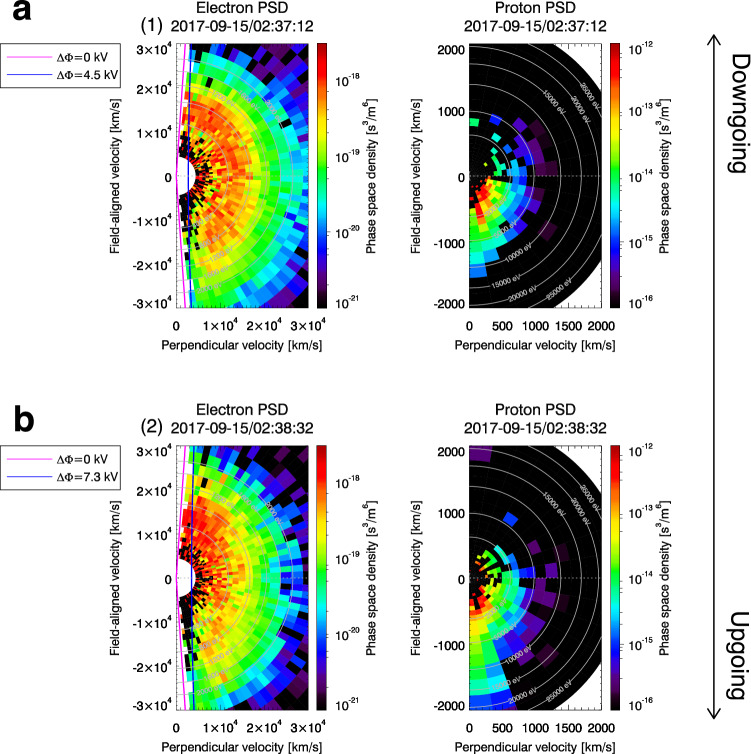
Snapshots of two-spin averaged phase space density (PSD) of auroral electrons and protons. Phase space density distributions of electrons and protons at: (**a**) 02:37:12 UT and (**b**) 02:38:32 UT which are labeled by (1) and (2) in Fig. 2, respectively. The positive field-aligned velocity indicates the velocity directed along the magnetic field vector (downward direction). The pitch angle intervals of electron and proton are 3° and 10°, respectively. The white region indicates no data in a pitch angle-energy bin. The magenta line shows the loss cone angle without a potential drop. The blue line shows the loss cone hyperbola in the presence of a potential drop estimated from the proton energy.

Figure 3 shows snapshots of the phase space density (PSD) distribution of auroral particles. The parallel electric field below the satellite expands the loss cone width and also renders it a function of energy (blue curve in Fig. 3a,b, left). During the event, there is a significant electron PSD within the loss cone in the downgoing direction (precipitating electron); however, there are very few electrons within the loss cone in the upgoing direction. This evidence indicates that accelerated electrons at very high altitudes indeed precipitate and power the visible aurora arc. The proton PSD that has a downward velocity component is quite small, indicating that few protons travelled to the satellite location from the magnetotail region (right panels in Fig. 3a,b); this suggests that most of these protons are repelled by the potential drop above the satellite.

The present observation cannot be explained by the conventional mechanisms of electron accelerations in the magnetotail, i.e., kinetic Alfvén waves and PSBL flows by tail reconnection. The time-varying parallel electric field by kinetic Alfvén waves, which have been reported also at high altitudes, contribute not to the monoenergetic acceleration, but the broadband-energy acceleration^27,28^. The magnitude of the Poynting flux of waves in the 3–180 s period range at the present event is only ~ 0.01 mW/m^2^, which is two orders smaller than the Alfvén wave events of previous observations at similar altitudes, because of the small magnetic perturbation (Supplementary Fig. S3). The PSBL electron beam, which is often associated with the magnetotail reconnection^29,30^, has a higher and broader energy range and shorter duration than our electron observations.

## Conclusions

**Figure 4:**
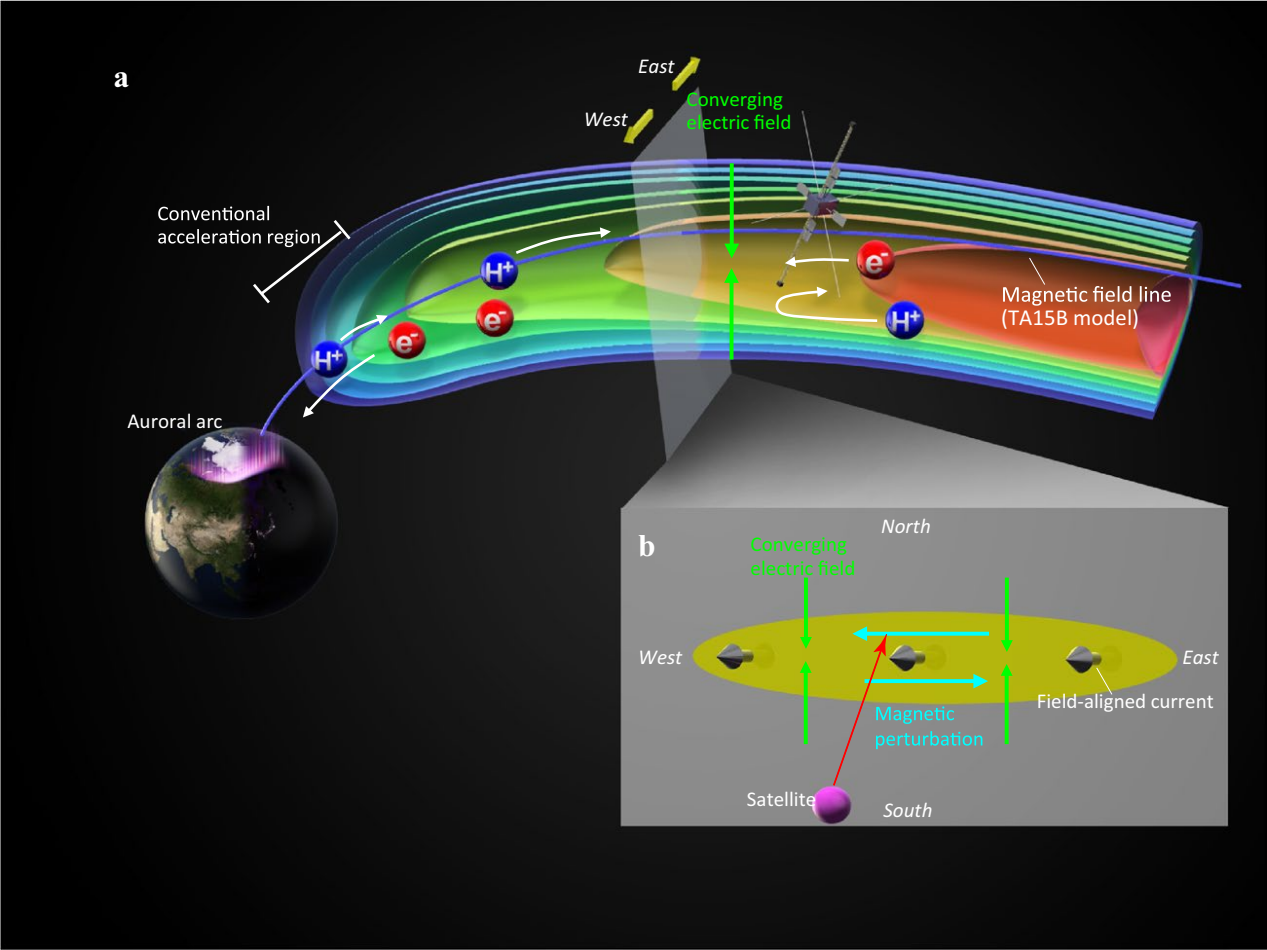
Schematic of the very high altitude electron acceleration powering an auroral arc. (**a**) Illustration in the meridional plane. (**b**) Illustration in the plane perpendicular to the background magnetic field.

Our observations agree with the typical characteristics of the acceleration region observed by previous low altitude satellites. This further compels us to modify our previous representation of the acceleration region^6^ and extend it to farther than the ~ 30,000 km altitude (Fig. 4) although the potential drop region along the field line above the satellite is not determined. The physical mechanism of the quasi-electrostatic parallel electric field is, however, still unknown. The double layer at the boundary between ionospheric and magnetospheric plasma has been considered as the location of the associated potential drop, residing at low-altitudes^31,32^. In the Earth’s radiation belts, near the magnetic equator, intense parallel electric fields are short-lived, narrow spatial structures^33^. This study demonstrates that the parallel electric field accelerating the auroral particle can exist at any height along a field line and is not limited to the transition region where the cold dense plasma from the ionosphere and the hot tenuous plasma from the magnetosphere coexist, suggesting some unknown magnetospheric mechanisms. Understanding the formation mechanism of the quasi-electrostatic parallel electric field is crucial for following the processes of discrete aurora emission and current transport on other planets, including Jupiter and Mars where potential-driven acceleration has been reported^34,35^.

## Methods

### Spin-tone noise reduction from the magnetic field

The present 64 Hz magnetic field data from Arase’s MGF instrument (Fig. 2f) include reduction of the spin-tone noise with an amplitude of several nT originating from the satellite spin with a period of ~ 8 s. Spin phase data $\theta_{s} \left( t \right)$ ($t$: time index) included in the MGF data product provide an accurate period of the spin-tone noise. The spin-tone noise can be modeled by a linear combination of sinusoidal functions $A = \left[ {\cos \theta_{s} \left( t \right),\sin \theta_{s} \left( t \right)} \right]$ and arbitrary parameters ${\varvec{x}} = \left[ {C_{1, } C_{2} } \right]^{T }$ that determine the amplitude and initial phase as, $A{\varvec{x}} = C_{1} \cos \theta_{s} \left( t \right) + C_{2} \sin \theta_{s} \left( t \right).$ We determine the parameters by minimizing the sum of the square differences between $A{\varvec{x}}$ and the 32-s high-pass filtered magnetic field data ${\varvec{B}}_{hp} \left( t \right)$ by the normal equation method, as ${\varvec{x}} = \left( {A^{T} A} \right)^{ - 1} A^{T} {\varvec{B}}_{hp}$. Because the parameters gradually vary with time, ${\varvec{x}}$ is calculated from data in a 10-min moving window with a time step of 10 min. Then the parameters are interpolated by the time index $t$. The model spin tone is produced from the spin phase data and the interpolated parameters. Finally, we subtract it from the original magnetic field data.

### Perpendicular electric field

The Arase’s EFD instrument observes the electric field in the plane perpendicular to the spin axis. The electric field parallel to the spin axis is complimented by the assumption of relatively small electric fields parallel to ${\varvec{B}}$, as ${\varvec{E}} \cdot {\varvec{B}} \approx 0$, where ${\varvec{E}}$ and ${\varvec{B}}$ are the electric and magnetic field vectors, respectively. The angle between ${\varvec{B}}$ and the spin plane was larger than 25° during the event, which is sufficiently large to use this technique. Then the background corotational and ${\varvec{v}}_{sc} \times {\varvec{B}}$ fields, where ${\varvec{v}}_{sc}$ is the satellite velocity vector, are subtracted from ${\varvec{E}}$. The two orthogonal components of the electric field perpendicular to ${\varvec{B}}$ are defined as follows: the east–west component is parallel to ${\varvec{B}} \times \user2{R }$ where ${\varvec{R}}$ is the outward radial position vector, and the poleward-equatorward component completes the orthogonal right-hand system.

### Loss cone hyperbola

The conservation of the first adiabatic invariant gives, $\frac{{v_{{M \bot^{2} }} }}{{B_{M} }} = \frac{{v_{{I \bot^{2} }} }}{{B_{I} }}$, where $B$ is the magnetic field strength, and $v$ is the particle velocity. Suffixes $I$ and $M$ donate values at the ionosphere (100 km altitude) and the magnetosphere (satellite location), respectively. The energy conservation of electrons barely reaching the ionosphere gives, $\frac{1}{2}m_{e} v_{I \bot }^{2} = \frac{1}{2}m_{e} (v_{M \bot }^{2} + v_{M\parallel }^{2} ) + e\Delta \Phi$, where $m_{e}$ is the electron mass, $e$ is the electron charge, and $\Delta \Phi$ is a potential difference. Combining the above equations, we have the loss cone hyperbola $v_{M \bot }^{2} \left( {\frac{{B_{I} }}{{B_{M} }} - 1} \right) - v_{M\parallel }^{2} = \frac{2e\Delta \Phi }{{m_{e} }}$ ^2^. In Fig. 3, we use $B_{M} = 260$ [nT] with the in-situ measurement and $B_{I} = 55500$ [nT] estimated by the International Geomagnetic Reference Field Model.

## Supplementary Information

Supplementary Video 1.

Supplementary Information 1.

## Data Availability

Data from Arase except for the high-angular resolution electron data used in this study are available from the ERG Science Center^18^ operated by ISAS/JAXA and ISEE/Nagoya University (https://ergsc.isee.nagoya-u.ac.jp/data_info/index.shtml.en). The present study analyzed L2 definitive orbit v03 data^37^, LEP-i L2 v03.00 data^38^ for protons, MGF L2 v03.03 64 Hz^39^ and spin-fit^40^ data for the magnetic field data, PWE/EFD L2 v03.00 data for the electric field data^41^ and v03.01 for the spacecraft potential data^42^. The pitch angle distribution of the high-angular resolution electron data is generated from fine channel data included LEP-e L1 v6 (calibrated, equivalent to L2) with MGF v03 data. THEMIS-ASI L1 v01 data and THEMIS ground magnetometers L2 v01 data (in Supplementary Fig. S2) are available at http://themis.ssl.berkeley.edu/data_all.shtml. Input parameters for magnetic field models are available at http://geo.phys.spbu.ru/~tsyganenko/modeling.html and https://omniweb.gsfc.nasa.gov/.

## Code availability

The software used to read and analyse the data, part of the publicly available SPEDAS software package including ERG plug-in tools (https://ergsc.isee.nagoya-u.ac.jp/analysis/spedas/index.shtml.en), is available at http://themis.ssl.berkeley.edu/software.shtml and can be used without any restriction. The publicly available IDL GEOPACK (v10.6) software (http://ampere.jhuapl.edu/code/idl_geopack.html) is used for calculating the model magnetic fields and performing field line tracings.
