# Supplementary material for: Active auroral arc powered by accelerated electrons from very high altitudes
Source: Sci Rep. 2021 Jan 18;11:1610. doi: 10.1038/s41598-020-79665-5 (PMC7814012; doi:10.1038/s41598-020-79665-5)
Supplement: Supplementary file 2 — Supplementary Information 1. [file 41598_2020_79665_MOESM2_ESM.docx]

**Supplementary Information**

**Title:**

**Active auroral arc powered by accelerated electrons from very high altitudes**

**Authors:** Shun Imajo^1^* (email: imajos@isee.nagoya-u.ac.jp), Yoshizumi Miyoshi^1^, Yoichi Kazama^2^, Kazushi Asamura^3^, Iku Shinohara^3^, Kazuo Shiokawa^1^, Yoshiya Kasahara^4^, Yasumasa Kasaba^5^, Ayako Matsuoka^6^, Shiang-Yu Wang^2^, Sunny W. Y. Tam^7^, Tzu‑Fang Chang^7^, Bo‑Jhou Wang^2^, Vassilis Angelopoulos^8^, Chae-Woo Jun^1^, Masafumi Shoji^1^, Satoko Nakamura^1^, Masahiro Kitahara^1^, Mariko Teramoto^9^, Satoshi Kurita^10^, Tomoaki Hori^1^

^1^Institute for Space-Earth Environmental Research, Nagoya University, Furo-cho, Chikusa-ku, Nagoya, Aichi, 464-8601, Japan.

^2^Academia Sinica Institute of Astronomy and Astrophysics, 11F Astronomy-Mathematics Building, AS/NTU, No. 1, Sec. 4, Roosevelt Road, Taipei 10617, Taiwan.

^3^Institute of Space and Astronautical Science, 3-1-1 Yoshinodai, Chuo-ku, Sagamihara, Kanagawa, 252-0222, Japan.

^4^Graduate School of Natural Science and Technology, Kanazawa University, Kakuma-machi, Kanazawa, Ishikawa, 920-1192, Japan.

^5^Graduate School of Science, Tohoku University, 6-3 Aoba, Aramaki, Aoba-ku, Sendai, 980-8578, Japan.

^6^Data Analysis Center for Geomagnetism and Space Magnetism, Graduate School of Science, Kyoto University, Oiwake-cho, Kitashirakawa, Sakyo-ku, Kyoto, 606-8502, Japan.

^7^ Institute of Space and Plasma Sciences, National Cheng Kung University, No.1, University Road, Tainan City 70101, Taiwan.

^8^Department of Earth, Planetary, and Space Sciences, University of California, Los Angeles, California 90095-1567, USA.

^9^Faculty of Engineering, Kyushu Institute of Technology, 1-1 Sensui-cho, Tobata-ku, Kitakyushu-shi, Fukuoka, 804-8550, Japan.

^10^Research Institute for Sustainable Humanosphere, Kyoto University, Gokasho, Uji City, Kyoto, 611-0011, Japan.

**List of Supplementary Information**

1. Supplementary Video S1. See the separated attached file.
2. Supplementary Figure S1. See below.
3. Supplementary Figure S2. See below.
4. Supplementary Figure S3. See below.


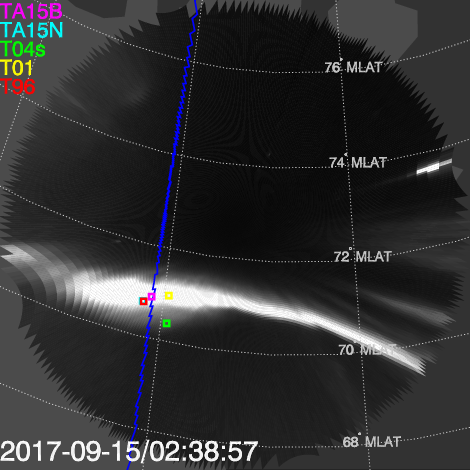


Supplementary Figure S1 | **Ionospheric footprint of Arase estimated by three magnetic field models.** Magenta, cyan, green, yellow, and red squares represent the footprint from various state-of-art magnetic field models; Tsyganenko and Andreeva (TA) 15B, TA 15N, T 04s, T 01, and T 96 models.

Supplementary Figure S2 | **Background situation of the Arase-arc conjunction event.** **a** Auroral intensity along the south-to-north cross-section at the blue line shown in Fig. 1. Magenta line indicates the magnetic latitude of Arase’s footprint. Arase observations of omni-directional differential energy fluxes of **b** electrons and **c** protons, and **d** residual east-west magnetic field. Magnetic field variations observed by THEMIS ground magnetometers^1^ at **e** RANK and **f** Gillam (GILL) in the local magnetic coordinate system (*HDZ* coordinates). The *H* component is local magnetic north, the *D* component is local magnetic east, and the *Z* component is downward. The ground magnetic field data are subtracted by values averaged by their values in the plotted interval. The motion of the auroral arc corresponds to the evolution of the auroral current system of the substorm inferred by Arase and ground magnetometers. The Arase satellite was located in the plasma sheet filled with one to several tens of keV particle populations before the arc crossing and in the magnetotail lobe with very low particle flux after the arc passed through the equatorial side.

**S**upplementary Figure S3 | **Activity of Alfvénic fluctuations in a 3–180 s period range. a** Magnetic field fluctuations. **b** Electric field fluctuations. **c** Field-aligned Poynting flux calculated from the magnetic and electric field fluctuations. The mean vector magnetic field was calculated from a 180 s running average of the locally measured magnetic field. In our event of 02:35–02:40 UT, the magnetic fluctuations were small (~ 1 nT), and the resulting field-aligned Poynting flux was only of the order of 0.01 mW/m^2^.
